# Separation of Amino Acids, Dyes, and Pigments Using Novel Pressurized Circular TLC Assembly for Secure Medical Imaging Applications

**DOI:** 10.1155/2023/9914633

**Published:** 2023-04-12

**Authors:** Amara Dar, Muhammad Nayab Ahmad, Ghufrana Samin, Muhammad Muzammil Jahangir, Rabia Rehman, Jamil Anwar, Zahrah T. Al-thagafi, Zelalem Meraf, Mustafa Musa Jaber

**Affiliations:** ^1^Centre for Analytical Chemistry, School of Chemistry, University of the Punjab, Quaid-e-Azam Campus, Lahore 54590, Pakistan; ^2^Department of Basic Sciences and Humanities, University of Engineering and Technology-Lahore, Faisalabad Campus, Faisalabad, Pakistan; ^3^Institute of Horticultural Sciences, University of Agriculture, Faisalabad, Pakistan; ^4^Centre for Inorganic Chemistry, School of Chemistry, University of the Punjab, Quaid-e-Azam Campus, Lahore-54590, Pakistan; ^5^Chemistry Department, University of Management & Technology, Lahore, Punjab, Pakistan; ^6^Department of Chemistry, College of Science, Taif University, P.O. Box 11099, Taif 21944, Saudi Arabia; ^7^Department of Statistics, Injibara University, Injibara, Ethiopia; ^8^Department of Medical Instruments Engineering Techniques, Al-Farahidi University, Baghdad 10021, Iraq

## Abstract

A novel pressurized flow system for circular thin-layer chromatography (PC-TLC) has been successfully established and employed for the separation of amino acids, dyes, and pigments for safe medical imaging applications. In this system, the mobile phase is applied to a regular TLC plate through the tube and needle of an intravenous infusion set. The needle was fused in a hole underneath the center of the plate, while the second side end of the tube was connected to a microburette containing the solvent. This new assembly proved itself better in terms of separation time (within 5 minutes) and controlled flow of the solvent and horizontal movement of analyte components over chromatograms with better separation and *R*_*f*_ values (glutamine: 0.26, valine: 0.44, phenylalanine: 0.60, chlorophyll a: 0.52, chlorophyll b: 0.43, xanthophyll: 0.18, carotenoid: 0.97, and pheophytin: 0.60) when a number of samples of amino acids, dyes, and pigments were separated by the developed apparatus and the conventional TLC procedure. The developed method was found distinctly rapid, precise, and eco-friendly (less solvent consuming) as compared to traditional ascending TLC.

## 1. Introduction

Thin-layer chromatography is one of the common classical chromatographic techniques used by analytical chemists to separate and identify different components of a complex sample. TLC has also been employed to evaluate the purity of a compound and monitor the progress of a reaction. High sensitivity, low cost, little separation time, and a wide range of applications are the factors which make this technique worldwide popular. It is a simple nonhazardous separation method which does not require any sophisticated instrumentation. Separation occurs due to the relative solubility of sample components in the mobile phase and the relative adsorption on the stationary phase, which is usually uniformly coated on a glass, plastic, or aluminum sheet. The most common adsorbents used for TLC are silica, alumina, and cellulose. A mobile phase is usually a single or mixture of two or more miscible solvents depending on the nature of the sample [1]. In ascending TLC, the liquid mobile phase is drawn up through the stationary phase by capillary action when, after loading the sample and standards, the adsorbent coated plate is vertically placed in a chromatographic tank. The mobile phase drags the sample components to different heights while travelling on the stationary phase. The separation of different components takes place on the basis of their retardation due to a stationary phase and solubility in the mobile phase [2]. Because of the importance of the separation of amino acids in TLC, different approaches have been reported for prediction of these retardation tendencies of such samples [3] like quantifiable structure-*R*_*f*_ connection of amino acids in various solvents for RP-TLC [4], structure-retardation factor relationship of amino acids in different mobile phases [5], and prediction of *R*_*f*_ of amino acids in RP-TLC with ethanol-sodium azide mixture as a mobile phase [6]. Different solvents were reported for variations in separation of colored components of leaf extracts [7].

A number of advancements in the classical TLC methodology [8] have been introduced since the pioneering work of Stahl in the early 1950s [9]. Some of the innovations improved the reproducibility, speed, and resolution of this technique as well as made it simpler and more cost-effective [10]. Improvements have also been made in the use of higher quality adsorbents along with the overpressure mobile phase. In this regard, reverse phase TLC and high-performance TLC are two examples [11]. Another is separating dyes with circular TLC [12]. One of the new ideas in TLC is to use a pressurized flow of the mobile phase over the adsorbent [5]. TLC with pressurized solvent flow, also known as “overpressure thin-layer chromatography” can be especially useful in high-performance TLC [13] where the plates are coated with finer particle adsorbents and the mobile phase travels more slowly to improve resolution [14]. The systems that are still used to apply the solvent under pressure are complex in design, expensive to purchase, cumbersome to organize, and difficult to operate. To get reproducible results from such systems is not an easy job for a nontechnical person. Our group already introduced a number of simple TLC activities for undergraduate students [15]. In the described procedure, the solvent is introduced in the center of the plate from a fine, controlled side burette through a thin plastic pipe. The solvent flows under gravity pressure and spreads on the adsorbent horizontally [16]. This system saved significant time and gave better resolution than classical TLC. The present system of feeding solvent to the plate is much simpler and faster than classical ascending TLC, making it perfect for high-performance TLC [17].

## 2. Experimental

### 2.1. Apparatus Assembly

**Figure 1:**
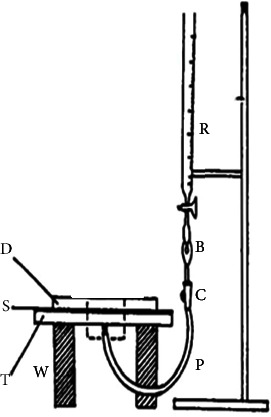
Apparatus assembly. B: dropper, C: wheel cock, D: Petri dish, S: TLC plate, P: plastic tubing, W: wooden blocks, T: glass plate, and R: burette.

Commercially available 10 × 10 inch TLC silica gel 60 F254 plates were used. A TLC plate was fixed on the same size glass plate and placed on the wooden blocks, as shown in Figure 1. A small hole (1 mm) was bored in the center of both plates. The needle and the pipe of an intravenous infusion set (model: Mediset, SF-35) was employed to deliver the solvent to the TLC plate. The needle was gently inserted in the central hole of the glass plate till its end reached the TLC plate, and the plastic pipe was connected to the outlet of the burette (model: Pyrex, 3B 846B) containing the solvent.

### 2.2. Chemicals Required

TLC silica gel 60 F254 plates (Merck KGaA), samples of amino acids (glutamine, valine, and phenylalanine), ninhydrin, inks (fountain pen ink and ballpoint ink), and ethanol and spinach leaves for leaf extracts were used in this study. All chemicals were of AnalR grade and obtained from Merck (Germany). Leaf extracts were prepared by crushing fresh leaves of spinach plants and extracting liquid from ethanol using Soxhlet apparatus [18].

### 2.3. PC-TLC Operating Setup

With the help of a 5 *μ*L micropipette, a sample spot was applied on a circle of 1 inch diameter on the midpoint of the plate. In order to saturate the system with solvent vapors, the spots were covered with a 10-inch Petri dish. After sampling, the flow-control valve of the burette was slowly opened to allow the solvent to flow over the plate. For the establishment of equilibrium, the solvent was not allowed to flow more than 0.2 mm/min over TLC. The flow of the mobile phase was organized primarily with the stopper at the end of the burette and second with the drop-falling valve mechanism of the infusion set. After adequate development, the plate was removed from the assembly, dried in an oven, and *R*_*f*_ values of colored bands were measured.

For the sake of comparison, samples of amino acids, inks, dyes, leaf extracts, and pigments were also analyzed by using classical ascending TLC. Strips (3 × 6 inches) of the same commercial plates and solvent composition were employed in the classical procedure. The separation and compound identification of leaf extracts have already been performed with the help of classical ascending TLC [19]. In this work, only the *R*_*f*_ values obtained for sample constituents, time consumed for separation, and resolution were compared by both methods.

### 2.4. Separation of Amino Acids

Amino acids such as phenylalanine, tryptophan, and valine [20] were separated by the described procedure of PC-TLC as well as by classical ascending TLC [21]. The solvent system used for the separation of amino acids consists of *n*-butanol, water, and acetic acid in a ratio of 60 : 25 : 15. Ninhydrin spray was employed to detect amino acids on the TLC plate [22].

### 2.5. Separation of Inks and Dyes

Inks used in fountain pens and ballpoints are usually the mixtures of various colored dyes and can be separated easily by TLC [1]. Both types of inks were used as samples in the present work. The solvent system used for the separation of ink ingredients consists of *n*-butanol, water, and acetic acid in a ratio of 60 : 25 : 15.

### 2.6. Separation of Pigments from Spinach Extracts

In this set of experiment, extracts of spinach [20] leaves were used as samples. The extracts were obtained from acetone by applying standard procedures [7] and analyzed by the described and classical TLC procedures. The mobile phase used for the separation of spinach extract ingredients was a mixture of hexane and acetone in a ratio of 7 : 3.

## 3. Results and Discussion

Pressurized circular TLC (PC-TLC) is an advanced form of circular TLC, which is also known as multidimensional TLC in the literature [16]. This PC-TLC assembly has been generally found to be simpler, faster, and more environmentally friendly than conventional ascending TLC. The new technique has numerous advantages over classical ascending TLC [12]. PC-TLC could be successfully employed for the separation of all types of colored samples with better resolution. The horizontal spreading of solvent in the case of PC-TLC takes less than half time for separation, giving better resolution. In addition, the solvent flowrate can be controlled through a stopper and valve system provided in the assembly. As compared to classical TLC, far less volume of solvent is required for achieving satisfactory separation. The use of relatively less volume of solvent makes the technique environmentally friendly. Another advantage which the newly described method offers is the provision of gradient elution which is not possible in classical TLC.

### 3.1. Amino Acids

**Figure 2:**
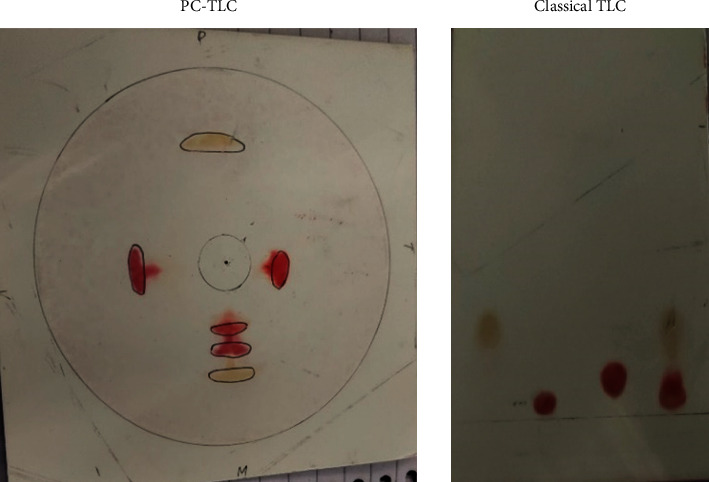
Amino acid separation by classical and pressurized TLC.

A synthetic mixture of three amino acids, phenylalanine, tryptophan, and valine, was analyzed by the newly described procedure and classical ascending TLC. The results obtained in both the techniques have been presented in Table 1, and separation is shown in Figure 2.

### 3.2. Inks and Dyes

**Figure 3:**
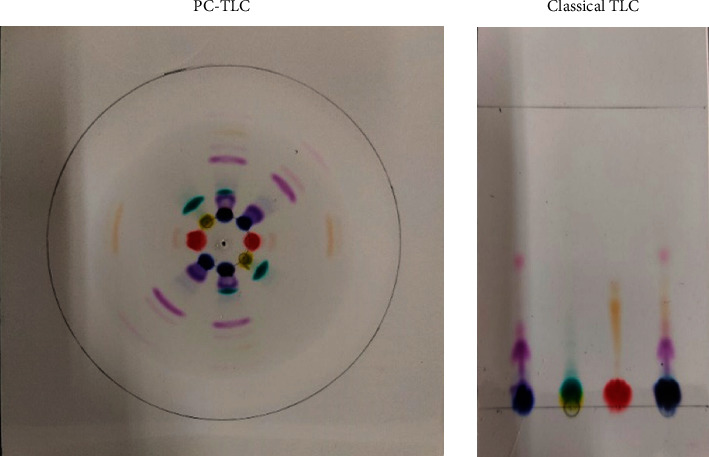
Fountain pen ink separation by classical and pressurized TLC.

**Figure 4:**
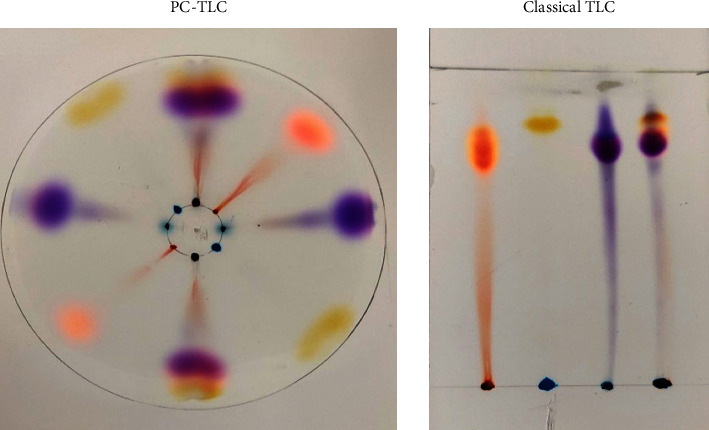
Ballpoint ink separation by classical and pressurized TLC.

Fountain pen ink and ballpoint ink are usually mixtures of various dyes which can be easily separated with the help of TLC. Both ink sample components were separated by classical and pressuized TLC. The *R*_*f*_ values and time consumed for separation of various components have been compared in Tables 2 and 3, and resolution is shown in Figures 3 and 4.

### 3.3. Pigments in Leaf Extracts

**Figure 5:**
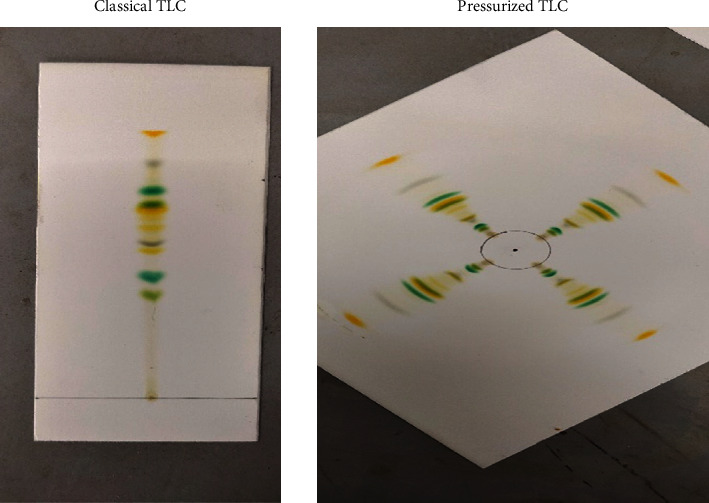
Separation of pigments from the spinach extract by classical and pressurized TLC.

Green leaves of plants contain several components which can be easily separated by TLC [20]. In this work, the extracts of spinach leaves have been analyzed by the described procedure of PC-TLC as well as by classical ascending TLC as used for beet root extract analysis [23]. The results of chromatographic analysis of the spinach extract are given in Table 4, and comparison of resolution of both method *R*_*f*_ has been shown in Figure 5, which indicated that almost the same *R*_*f*_ values resulted with PC-TLC within 10 minutes as compared to conventional methods.

## 4. Conclusion

Pressurized circular TLC (PC-TLC) is a relatively simple, fast, and cost-effective technique of TLC, which can yield better resolution of all types of samples which can be analyzed by classical ascending thin-layer chromatography for medical imaging applications. As compared to normal TLC, the new method consumes almost half the time (within 5 minutes) for obtaining even better results. This technique needs far less volume of solvent for separation and hence can be claimed as cost-effective as well as environmentally friendly. In addition to other advantages, gradient elution can conveniently be incorporated in the described procedure, whereas this facility is not possible in normal ascending TLC.

## Figures and Tables

**Table 1:** Separation of amino acids by classical and pressurized TLC.

Systems used for separation	Time consumed (min)	*R*_*f*_ values obtained for different amino acids		
		Glutamine	Valine	Phenylalanine
Ascending TLC	19	0.25	0.43	0.61
PC-TLC	5	0.26	0.44	0.60

**Table 2:** Separation of fountain pen ink by classical and pressurized TLC.

Systems used for separation	Time consumed (min)	*R*_*f*_ values obtained for different components		
		Red	Blue	Green
Ascending TLC	20	0.77	0.84	0.79
PC-TLC	5	0.81	0.88	0.84

**Table 3:** Separation of ballpoint ink by classical and pressurized TLC.

Systems used for separation	Time consumed (min)	*R*_*f*_ values obtained for different components		
		Red	Blue	Green
Ascending TLC	20	0.55	0.24	0.67
PC-TLC	5	0.57	0.26	0.68

**Table 4:** Separation of pigments from the extract of spinach leaves by both methods.

Systems used for separation	Time taken (min)	*R*_*f*_ values obtained for different pigments present in the spinach extract				
		Chlorophyll a	Chlorophyll b	Xanthophyll	Carotenoid	Pheophytin
Classical TLC	18	0.52	0.42	0.17	0.98	0.59
PC-TLC	5	0.52	0.43	0.18	0.97	0.60

## Data Availability

All data related to this work are presented in the results section along with references.
